# Supplementary material for: Development and evaluation of an artificial intelligence system for COVID-19 diagnosis
Source: Nat Commun. 2020 Oct 9;11:5088. doi: 10.1038/s41467-020-18685-1 (PMC7547659; doi:10.1038/s41467-020-18685-1)
Supplement: Supplementary file 1 — Supplementary Information [file 41467_2020_18685_MOESM1_ESM.pdf]

Supplementary information

Development and Evaluation of an Artificial Intelligence System for COVID-19 Diagnosis

Cheng Jin, Weixiang Chen, Yukun Cao, Zhanwei Xu, Zimeng Tan, Xin Zhang, Lei Deng, Chuansheng Zheng, Jie Zhou, Heshui Shi, and Jianjiang Feng

Supplementary methods

Data Pre-processing. The CT data in COVID-19 database were collected by different scanners in three different centers in Wuhan, so that the spacings of them are different. For a better performance of our proposed deep learning method, a resampling process was done to regularize them to 1×1×1 mm per voxel. The HU value of CT volumes ranges from -2048 to 3071. For a clear view of lungs and tissues inside or around lungs, we truncated a window of [-1200,700], and normalized the value to float value ranging from 0 to 1.

Slice Extraction. Our COVID-19 slice diagnosis network uses two-dimensional slices in axis direction instead of three-dimensional volumes as input.

For training of slice diagnosis network, slices were sampled from CTs of four different classes. For training cohort, all CTs were extracted to slices offline for training, while for test cohort, the whole CT volumes were feed to the AI system. We firstly sampled all cases in training cohort in axis direction within the lung. After slice extraction, we collected 282,805 slices for training consisting of 90,728 healthy slices, 45,476 CAP slices, 1,760 influenza-A/B slices and 144,571 COVID-19 slices.

For test of slice diagnosis network, we use all slices in the whole volume. Our system will automatically swap and compute on every slice and output the case level results via task-specific fusion module.

For training and test of COVID-infectious slice locating network, slices were only extracted from COVID-19 cases. We manually annotated COVID-infectious slice on 152 COVID-19 cases, of which 72 were from training cohort and 80 were from test cohort. According to the manual annotation, we finally get 3,758 slices of training samples (1,135 abnormal slices and 2,623 normal slices) and 4,707 slices of test samples (1,684 abnormal slices and 3,023 normal slices) for COVID-infectious slice locating.

Lung Segmentation Network. We have done transfer learning based on a widely used segmentation model UNet. This network can process two-dimensional images and produce binary segmentation maps. We used 100 COVID-19 CTs in the training cohort with annotated lung mask to train the lung segmentation network and their lung areas were manually annotated. CT volumes were cut into slices and 16,223 slices were obtained (Supplementary Figure 1). We found that segmentation network trained on COVID-19 cases was able to segment CTs of other categories well.

Slice Diagnosis Network. The network structure is ResNet152, a deep network with 152 convolutional, pooling or fully-connected layers. The input of this part is lung masked images, whose three channels were respectively raw image, raw image dot-times lung mask, lung mask. The input images were cropped along the bounding box of lung masks. Then, all input slices are resized to 224x224 and fed to network (Supplementary Figure 2 a b). We have tested three different classification networks before choosing the proposed one, including 3D classification network, 2D

classification network with case-level supervision (also called week supervision). Supplementary Figure 3 shows the structures of them. Besides a naïve method of the proposed network, which uses input image without segmentation mask of lung, has been tested. All these three different networks performed worse than the proposed one.

COVID-infectious Slice Locating Network. We fine-turned our slice diagnosis network to get the COVID-infectious slice locating network. This network was trained for 20 epochs.

CXR diagnosis Network. We exploited the convolutional neural network to distinguish COVID-19 and CAP cases from CXR scans. The CXR images fed to the network were cropped along the lung area and resized to 512x512 first. The system will learn the context information automatically and decide the probabilities that the input falls into two categories. Our network consists of 4 convolutional layers followed by max-pooling layers, and 2 fully-connected layers (Supplementary Figure 6). This network was trained for 150 epochs. Considering the small proportion of CAP in the training images, we applied data augmentation.

Choice of K. We performed a 4-fold cross-validation on the training cohort to find the best K in top-K fusion. Other than top-K fusion, we also tested results using NO. K method, which uses the K-th highest score of every category. Both fusion methods are designed for reducing false positives. Performances are listed in Supplementary Table 1 and the metric is averaged accuracy.

Supplementary Table 1 | Accuracy of different fusion settings.

Method	K	Accuracy
Top-K	1	0.8755 (0.8705-0.8806)
	3	0.8921 (0.8872-0.8966)
	5	0.8863 (0.8826-0.8915)
	7	0.8921 (0.8868-0.8970)
NO. K	2	0.8752 (0.8712-0.8794)
	5	0.8853 (0.8818-0.8904)

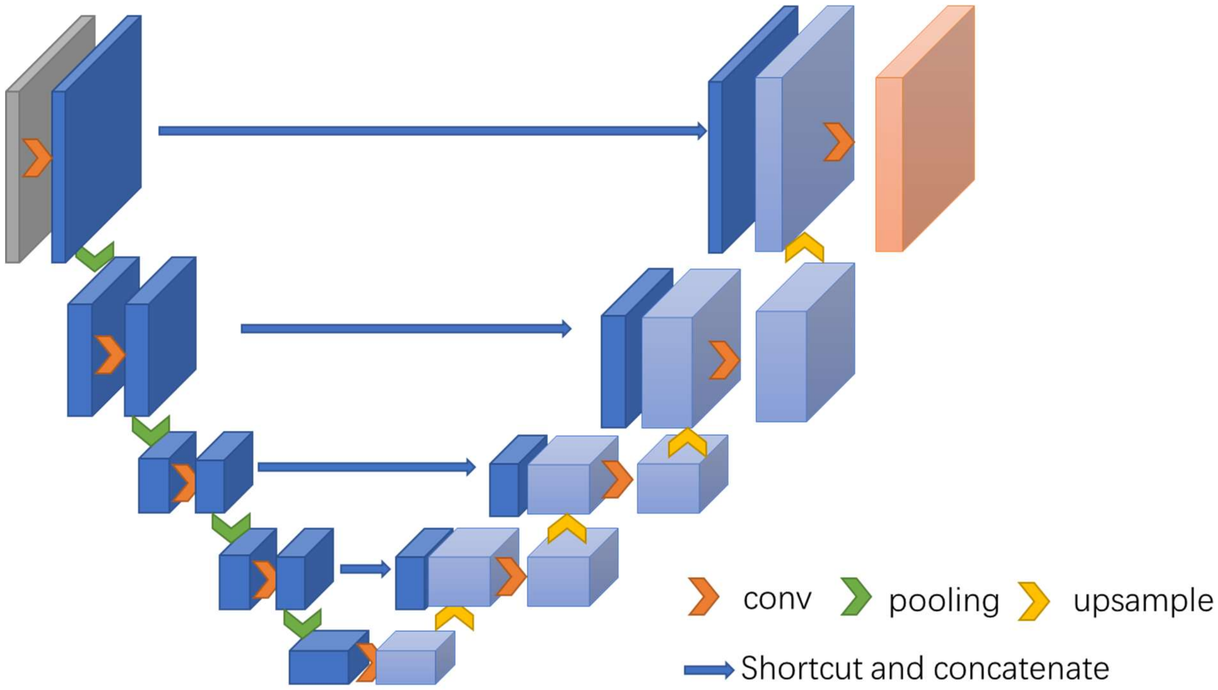

Supplementary Figure 1 | Network structure of lung segmentation network is Unet.

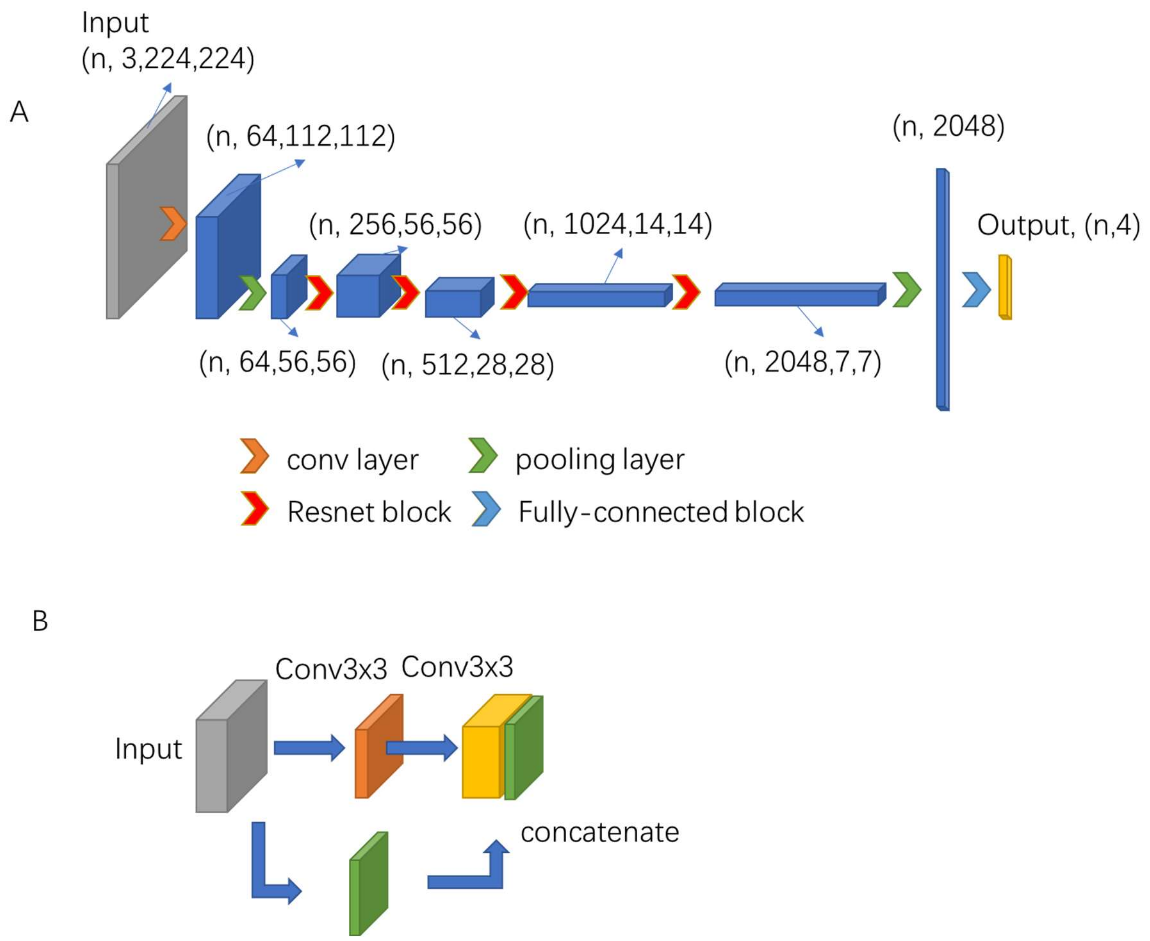

Supplementary Figure 2 | Structure of ResNet152, the slice diagnosis network. a. The overview of structure of ResNet152. The basic block of this network is notated as Resnet block. b. Structure of Resnet blocks.

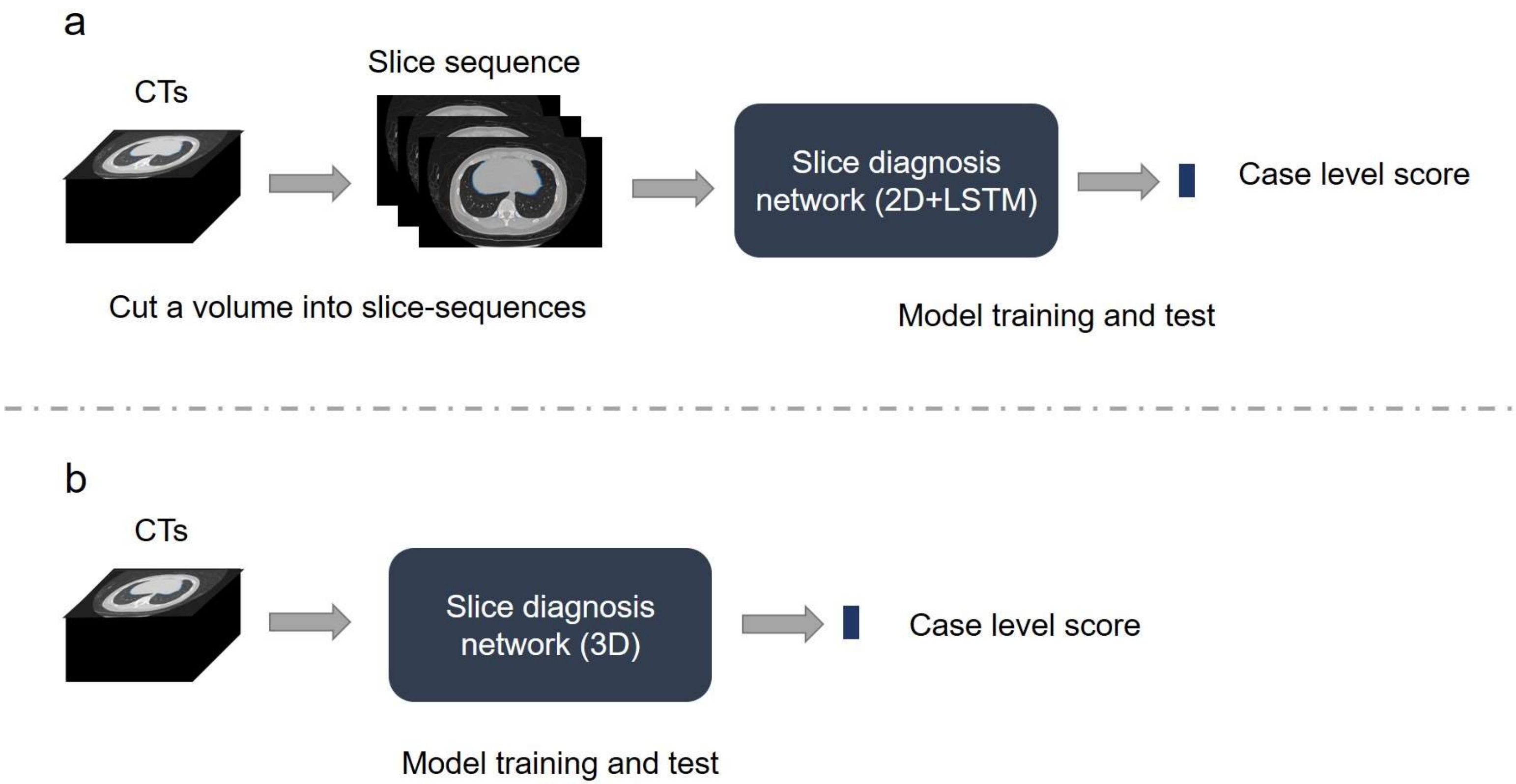

Supplementary Figure 3 | Workflows of the two other methods which have been proved weaker than the proposed slice diagnosis network by experiments. a. workflow of 2D method with weak supervision at case level. **b.** workflow of 3D method with weak supervision at case level.

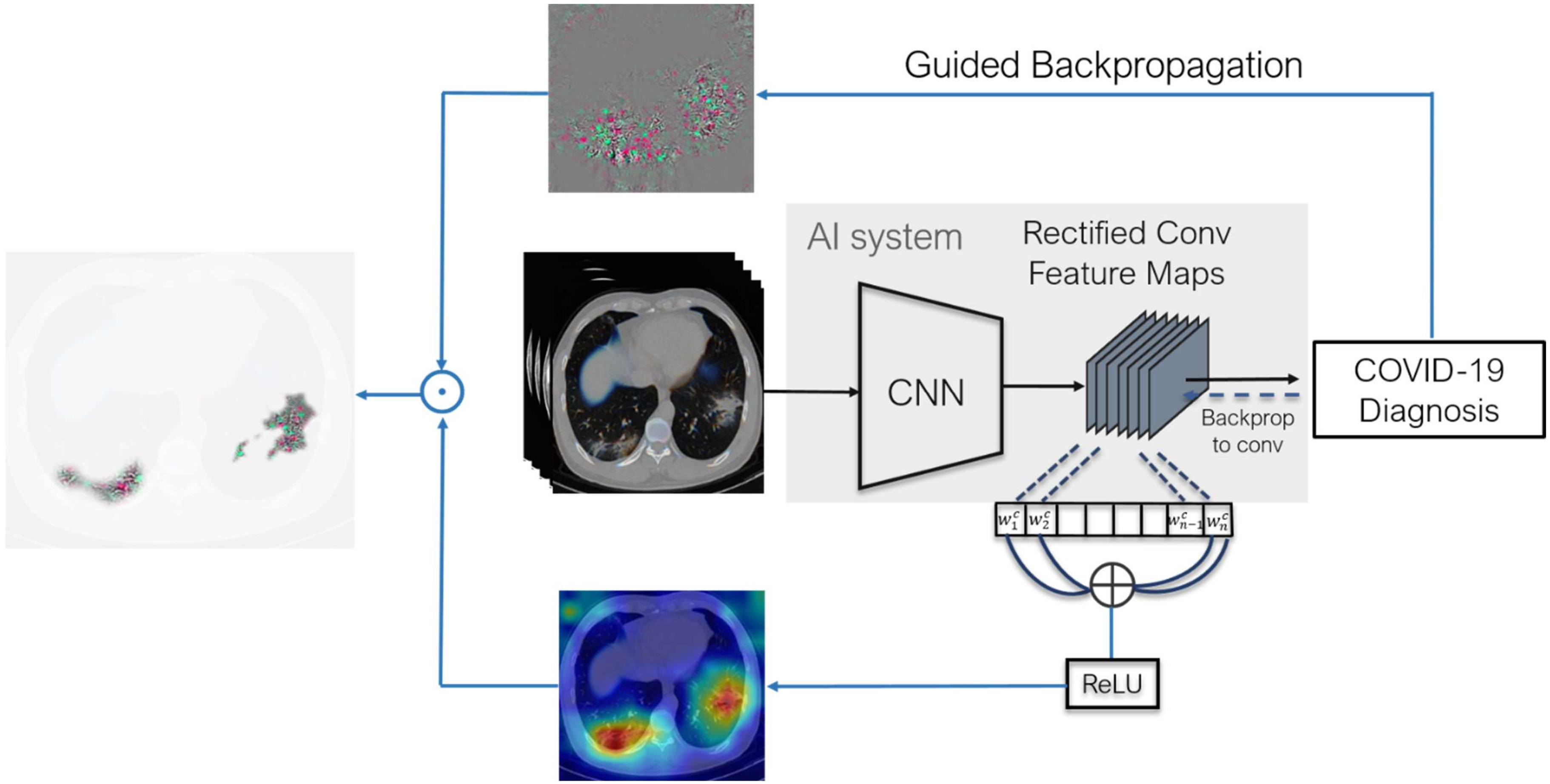

Supplementary Figure 4 | Structure of visualization module.

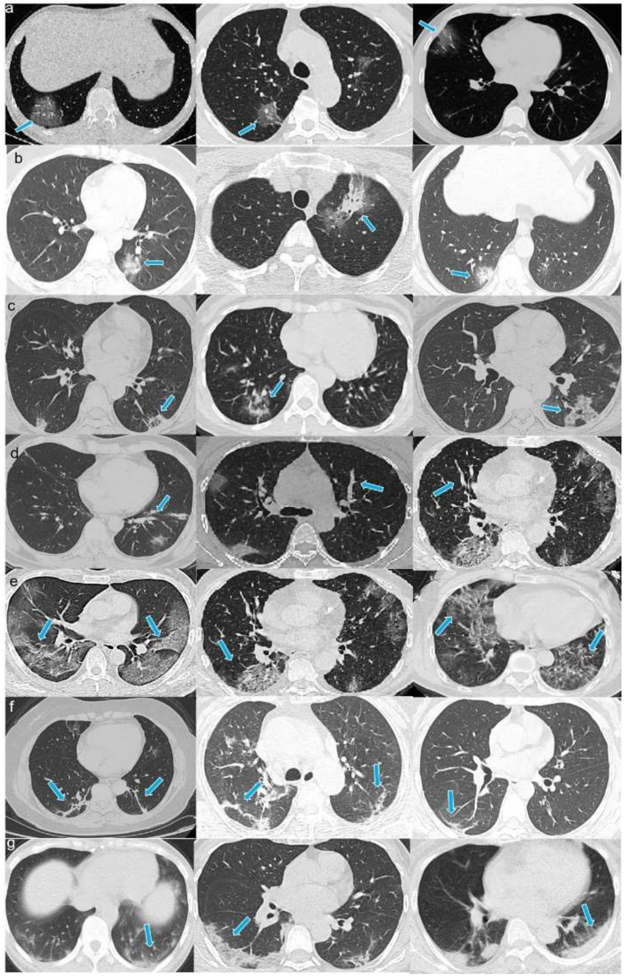

Supplementary Figure 5 | Some typical CT imaging signs of COVID-19. a. Spherical ground-glass opacity, **b.** Halo, **c.** Anti-halo, **d.** Enlarged vascular, **e.** Crazy paving, **f.** Cord, **g.** Band. (lesions marked by blue arrows)

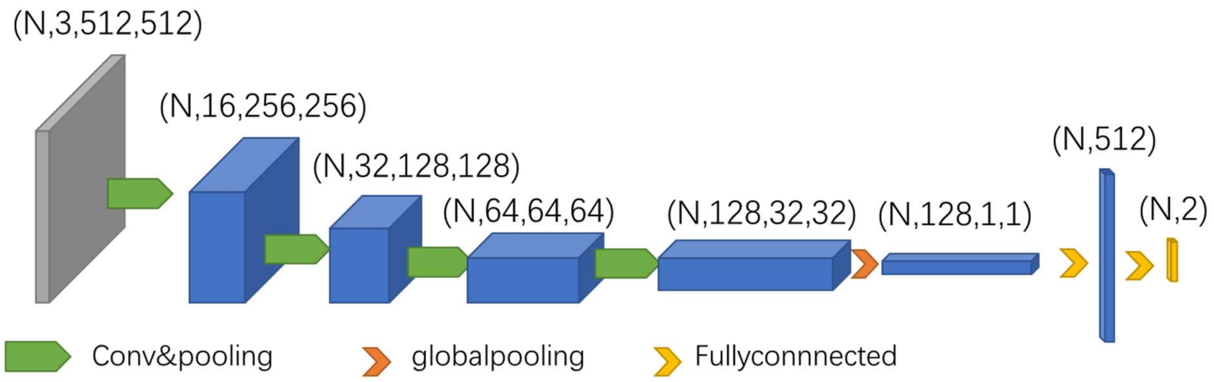

Supplementary Figure 6 | Structure of the CXR-based COVID-19 diagnosis network.

A

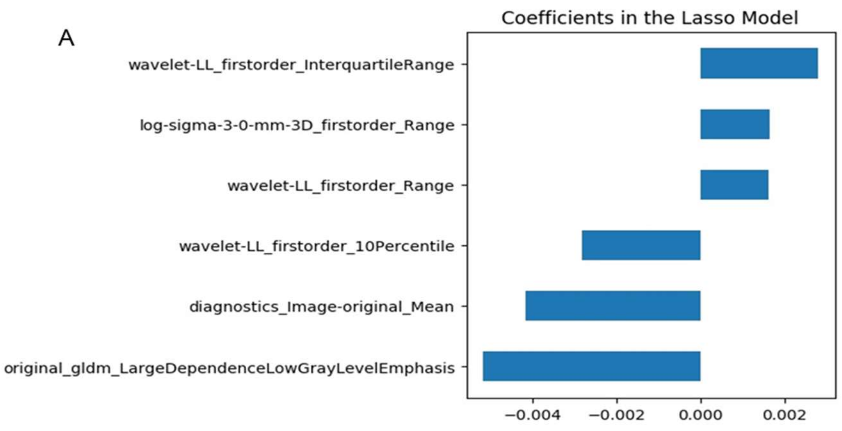

Feature	Coeffs.	P value of Kolmogorov-Smirnov test	P value of t test
wavelet-LL_firstorder_Range	0.00161	1	5.87945E-06
log-sigma-3-0-mm-3D_firstorder_Range	0.00162	1	2.99261E-36
wavelet-LL_firstorder_InterquartileRange	0.00278	1	9.53012E-24
wavelet-LL_firstorder_10Percentile	-0.00283	4.85202E-91	5.4302E-180
diagnostics_Image-original_Mean	-0.00418	5.4944E-140	3.5915E-185
original_gldm_LargeDependenceLowGrayLevelEmphasis	-0.00517	1	0.000933499
R-Score		1	1.63248E-60

B

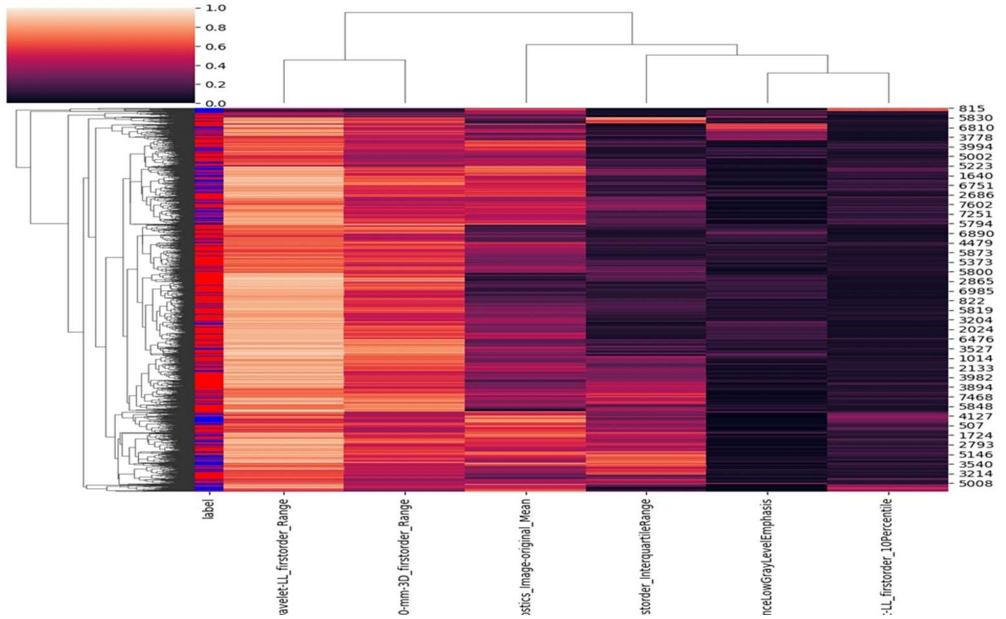

Supplementary Figure 7 | Selected radiomics features to identify COVID-19 from CAP. A. Coefficients of selected features and the R-score provided by LASSO model based on those features. Kolmogorov-Smirnov test and two-side t test were also done. **B.** Cluster heatmap of the selected features.

A

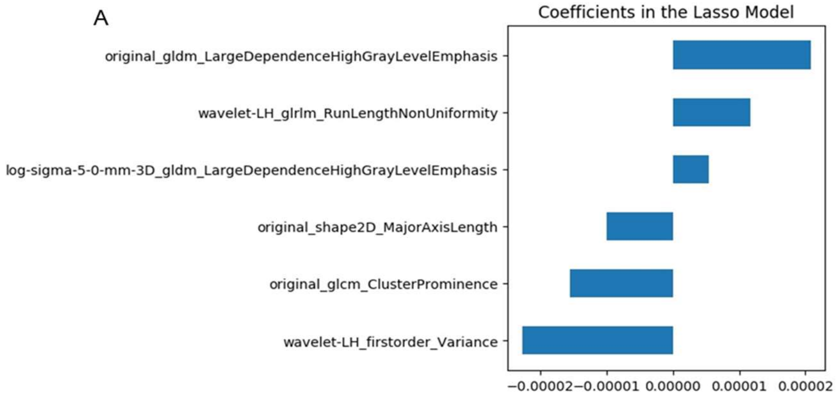

Feature	Coeffs.	P value of Kolmogorov-Smirnov test	P value of t test
wavelet-LH_firstorder_Variance	-2.27162E-05	0.001344905	5.18408E-07
original_glcm_ClusterProminence	-1.54831E-05	0.208421573	0.170687395
original_shape2D_MajorAxisLength	-9.9763E-06	0.556709296	0.316682052
log-sigma-5-0-mm-3D_gldm_LargeDependenceHighGrayLevelEmphasis	5.37301E-06	0.01990565	0.037690662
wavelet-LH_glrlm_RunLengthNonUniformity	1.17182E-05	0.68483196	0.324839758
original_gldm_LargeDependenceHighGrayLevelEmphasis	2.07946E-05	0.038355636	0.156599451
R-Score	-	0.011487257	0.035044266

B

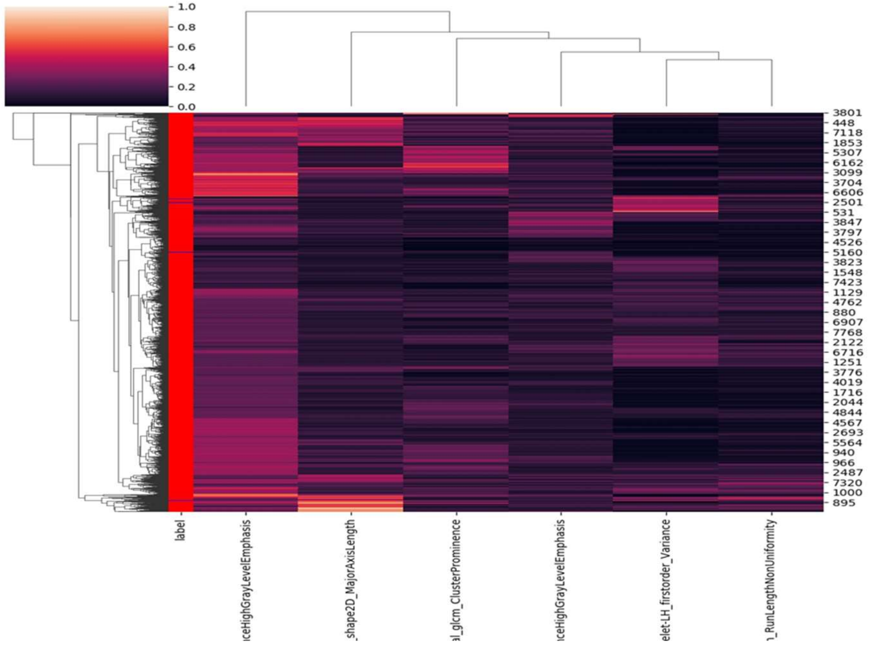

Supplementary Figure 8 | Selected radiomics features to identify COVID-19 from influenza-A/B. A. Coefficients of selected features and the R-score provided by LASSO model based on those features. Kolmogorov-Smirnov test and two-side t test were also done. **B.** Cluster heatmap of the selected features.

A B C

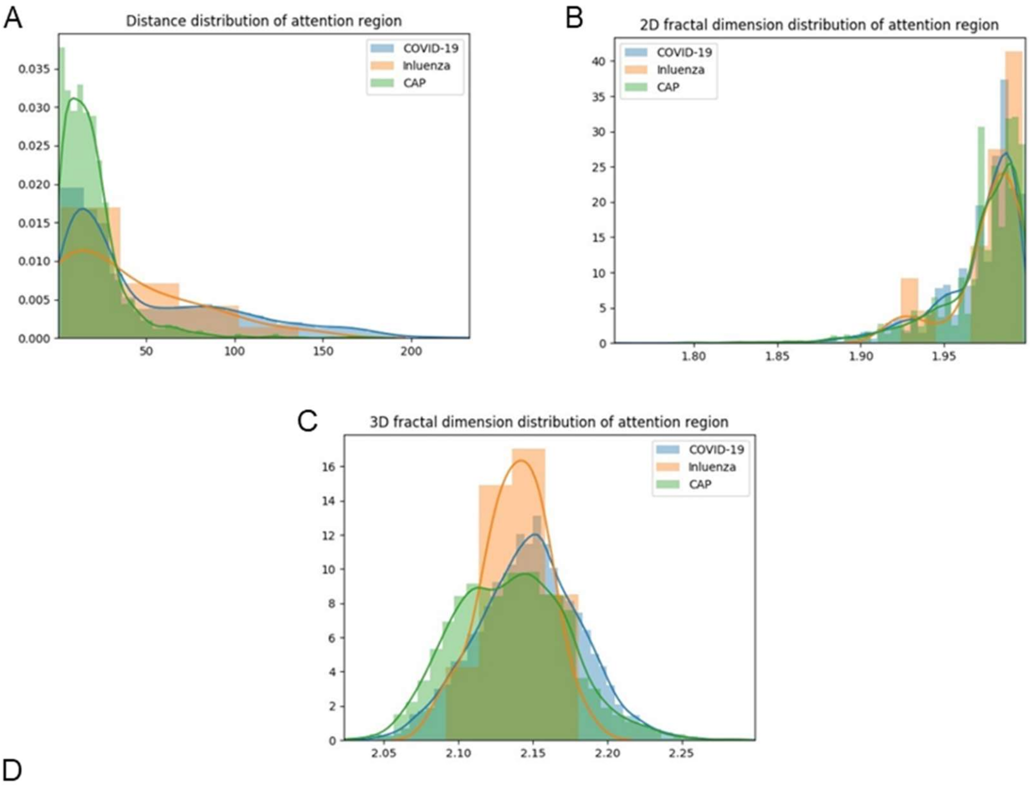

D

Feature	Compare with	P of Kolmogorov-Smirnov test	P of t test
Distance	CAP	**5.1079E-140**	**1.3669E-165**
Distance	Influenza	0.360381926	0.270528569
2D fractal	CAP	1	0.008808678
2D fractal	Influenza	0.442364417	0.260897171
3D fractal	CAP	1	**6.74069E-38**
3D fractal	Influenza	0.049154366	0.221171968

Supplementary Figure 9 | Distribution of three attentional region related features. A. Distances from the center of attentional region to lung's margin. **B.** Margin fractal dimension of attentional region. **C.** Gray level mesh fractal dimension off attentional region. **D.** P value of Kolmogorov-Smirnov test and two-side t test of three features between COVID-19 and CAP or influenza.
