# Supplementary material for: Development and evaluation of an artificial intelligence system for COVID-19 diagnosis
Source: Nat Commun. 2020 Oct 9;11:5088. doi: 10.1038/s41467-020-18685-1 (PMC7547659; doi:10.1038/s41467-020-18685-1)
Supplement: Supplementary file 2 — Reporting Summary [file 41467_2020_18685_MOESM2_ESM.pdf]

nature research

Corresponding author(s): Jianjiang Feng, Heshui Shi

Last updated by author(s): Sep 2, 2020

Reporting Summary

Nature Research wishes to improve the reproducibility of the work that we publish. This form provides structure for consistency and transparency in reporting. For further information on Nature Research policies, see Authors & Referees and the Editorial Policy Checklist .

Statistics

For all statistical analyses, confirm that the following items are present in the figure legend, table legend, main text, or Methods section.

n/a	Confirmed	
☐	☒	The exact sample size (*n*) for each experimental group/condition, given as a discrete number and unit of measurement
☐	☒	A statement on whether measurements were taken from distinct samples or whether the same sample was measured repeatedly
☐	☒	The statistical test(s) used AND whether they are one- or two-sided<br>*Only common tests should be described solely by name; describe more complex techniques in the Methods section.*
☒	☐	A description of all covariates tested
☐	☒	A description of any assumptions or corrections, such as tests of normality and adjustment for multiple comparisons
☐	☒	A full description of the statistical parameters including central tendency (e.g. means) or other basic estimates (e.g. regression coefficient) AND variation (e.g. standard deviation) or associated estimates of uncertainty (e.g. confidence intervals)
☐	☒	For null hypothesis testing, the test statistic (e.g. *F*, *t*, *r*) with confidence intervals, effect sizes, degrees of freedom and *P* value noted<br>*Give P values as exact values whenever suitable.*
☒	☐	For Bayesian analysis, information on the choice of priors and Markov chain Monte Carlo settings
☒	☐	For hierarchical and complex designs, identification of the appropriate level for tests and full reporting of outcomes
☒	☐	Estimates of effect sizes (e.g. Cohen's *d*, Pearson's *r*), indicating how they were calculated

Our web collection on statistics for biologists contains articles on many of the points above.

Software and code

Policy information about availability of computer code

Data collection	Dicom files were handled with the open source library Pydicom (https://pydicom.github.io/, version v1.4.2). Preprocessing was done using SimpleITK (http://www.simpleitk.org/, version 1.2.4). Slicer 4.10.2 was used to visualize volume in reader study.
Data analysis	Data analysis was conducted in Python (version 3.6) using the numpy (version v1.15.3), pyradiomics (version 2.2.0), scipy (version 1.3.3), and scikit-learn (version 0.22) packages. Deep learning method was implemented in open source repositories Pytorch (https://pytorch.org/, version 1.3.1) and torchvision (version 0.4.2). All codes related to diagnosis are available at https://github.com/ChenWWWeixiang/diagnosis_covid19

For manuscripts utilizing custom algorithms or software that are central to the research but not yet described in published literature, software must be made available to editors/reviewers. We strongly encourage code deposition in a community repository (e.g. GitHub). See the Nature Research guidelines for submitting code & software for further information.

Data

Policy information about availability of data

All manuscripts must include a data availability statement. This statement should provide the following information, where applicable:

- Accession codes, unique identifiers, or web links for publicly available datasets
- A list of figures that have associated raw data
- A description of any restrictions on data availability

Four databases in our experiments are publicly available. LIDC-IDRI database can be accessed at https://wiki.cancerimagingarchive.net/display/Public/LIDC-IDRI. Tianchi-Alibaba database can be accessed at the webpage (https://tianchi.aliyun.com/competition/entrance/231601/information?lang=en-us) of the challenge after registration. CC-CCII database can be accessed at http://ncov-ai.big.ac.cn/download. MosMedData database can be accessed at https://mosmed.ai/en/.
The datasets from Wuhan Union Hospital, Western Campus of Wuhan Union Hospital, and Jianghan Mobile Cabin Hospital were used under the license of the current study from Union Hospital, Tongji Medical College, Huazhong University of Science and Technology, Wuhan, China (2020/0030), so they are not publicly available. Interestinged readers may contact Heshui Shi for further information about these datasets.

Field-specific reporting

Please select the one below that is the best fit for your research. If you are not sure, read the appropriate sections before making your selection.

- [x] Life sciences
- [ ] Behavioural & social sciences
- [ ] Ecological, evolutionary & environmental sciences

For a reference copy of the document with all sections, see nature.com/documents/nr-reporting-summary-flat.pdf

Life sciences study design

All studies must disclose on these points even when the disclosure is negative.

Sample size

We collected data from both hospitals in Wuhan and three publicly available databases.
Under institution review board (IRB) approval, we collected data from three centers in Wuhan, which are Wuhan Union Hospital, Western Campus of Wuhan Union Hospital, and Jianghan Mobile Cabin Hospital. CT volumes of COVID-19 patients from Wuhan were collected from February 5th, 2020 to March 29th, 2020, and all those patients were confirmed as COVID-19 by RT-PCR. Heathy subjects came from physical examinations of Union Hospital from January 2nd, 2020 to February 2nd, 2020 and subjects with lung disease were excluded. CAP volumes were collected from January, 2019 to November, 2019. Influenza-A/B volumes were collected from November, 2016 to November, 2019. All CAP and influenza subjects were retrospective and confirmed subjects which must not be COVID-19 according to study dates. In total, 4,260 CT scans (2,529 COVID-19 scans, 1,338 CAP scans, 135 influenza-A/B scans and 258 normal scans) from 3,177 patients (1,502 COVID-19 patients, 83 influenza-A/B patients, 1,334 CAP patients, and 258 normal people) were collected from multi-centers.

LIDC-IDRI and Tianchi-Alibaba are both databases for lung nodule detection with separately 1,009 and 1,200 scans available. All subjects of them suffered from benign or malignant lung nodules. Because nodules have totally different presentations, we setup a category “non-pneumonia” to cover both healthy subjects from Wuhan and subjects from LIDC-IDRI and Tianchi-Alibaba. All above data was randomly divided into two independent parts with no overlapping subjects. The ratio of division is 1:1 that 2,688 subjects (3,263 scans) were assigned to training cohort which contained 1,230 non-pneumonia, 666 CAP, 41 influenza-A/B and 751 COVID-19 subjects were in training cohort while 2,690 subjects (3,203 scans) were assigned to test cohort which contained 1,229 non-pneumonia subjects, 668 CAP, 42 influenza-A/B and 751 COVID-19 subjects test cohort.

CC-CCII database shares only the image slices and some unknown processes had been performed to extract these slices from CT volumes. Besides, some of the slices are raw image slices while some others are cropped by lung masks. Its category definition is also a little different from ours. We used this database as an external test set which has 2,539 subjects (3,784 scans) .

CT scans of MosMedData database were obtained between 1st of March, 2020 and 25th of April, 2020, and provided by municipal hospitals in Moscow, Russia. We used this database as another external test set which has 1,110 subjects .

Data exclusions

For data from hospitals in Wuhan, we collected chest CTs with resolutions of 1.25mm or 5mm and detailed reliable medical reports. We excluded 11 cases which have obvious artifacts because of moving or breathing that influenced human radiologists' reading, but cases with slight artifacts due to moving or breathing were not excluded. For patients of COVID-19 with multiple CT scans, the last CT scan may be performed when patients are rehabilitative. To avoid noisy groundtruth, we did not use the last CT scans of patients with multiple CT scans. All exclusions were pre-established before experiments.

Replication

Our deep learning model converges with proposed hyper-parameters in different initialization and training data iteration order. Our test cohort and reader study cohort were independent with each other and independent with training cohorts. Performances of our system on both cohorts were consistent. We have replicated the results twice and the results of the three tests were almost the same. Since deep learning methods might converge differently at replicated experiments, we can only ensure that the replication results will not go beyond the confidence intervals reported.

Randomization

Subjects were randomized into training, test and reader study cohorts by shuffle function of python toolkit numpy with no overlapping at subject level.

Blinding

The deep learning models were developed on the training cohort. Hyper-parameters of the models were chosen according to performances on the training cohort. All training was done without any information from the test cohort and the reader study cohort. Models were tested on the test cohort and reader study cohort with fixed parameters.

Reporting for specific materials, systems and methods

We require information from authors about some types of materials, experimental systems and methods used in many studies. Here, indicate whether each material, system or method listed is relevant to your study. If you are not sure if a list item applies to your research, read the appropriate section before selecting a response.

Materials & experimental systems

n/a	Involved in the study
☒	☐ Antibodies
☒	☐ Eukaryotic cell lines
☒	☐ Palaeontology
☒	☐ Animals and other organisms
☐	☒ Human research participants
☒	☐ Clinical data

Methods

n/a	Involved in the study
☒	☐ ChIP-seq
☒	☐ Flow cytometry
☒	☐ MRI-based neuroimaging

Human research participants

Policy information about studies involving human research participants

Population characteristics	Under institution review board (IRB) approval, we collected data from three centers in Wuhan, which are Wuhan Union Hospital, Western Campus of Wuhan Union Hospital, and Jianghan Mobile Cabin Hospital. 1,502 COVID-19 patients were collected from February 5th, 2020 to March 29th, 2020; 83 influenza-A/B patients were collected from November, 2016 to November, 2019; 1,334 CAP patients were collected from January, 2019 to November, 2019; 258 healthy normal people were collected from January 2nd, 2020 to February 2nd, 2020. For healthy cases, 59 were male, 199 were female and they aged from 19 to 64 (mostly between 21-40); for CAP cases, 824 were male and 506 were female, and they aged from 17 to 89 (mostly between 61-80); for influenza cases, 54 were male and 29 were female, and they aged from 20 to 88 (mostly between 61-80); for COVID-19 cases, 717 were male and 785 were female, and they aged from 17 to 85 (mostly between 61-80).<br><br>Characteristics for publicly available databases can be found on their websites, but most of characteristics have been removed for anonymization.
Recruitment	The COVID-19 data were gathered retrospectively from three centers in Wuhan, China, which is a major epidemic area in China. We first include all available data in the three centers during February 5th, 2020 to March 29th, 2020 and excluded some of them according to the rules stated in data exclusions parts. Wuhan Union Hospital, Western Campus of Wuhan Union Hospital treated normal and severe patients at that time while Jianghan Mobile Cabin Hospital treats only mild patients, so both normal, mild and severe patients were gathered. Influenza, CAP cases were followed the same procession that no selection bias was introduced. Healthy normal cases were all health examination cases of Wuhan Union Hospital and most of them are younger than patients mentioned above. We added LIDC-IDRI and Alibaba-Tianchi into experiments and mixed them with normal cases as non-pneumonia group to decrease this age bias.<br><br>Since all patients were from Wuhan, China, some bias for races might be introduced. Concerned about this issue, a publicly available database from Russia, MosMedData, was used as an independent test cohort.
Ethics oversight	Data collection in Wuhan received ethical approval from Union Hospital, Tongji Medical College, Huazhong University of Science and Technology, Wuhan, China (2020/0030). As a retrospective stduy, the need for informed consent was waived by the institutional review board.

Note that full information on the approval of the study protocol must also be provided in the manuscript.
